# Real intensity of physical activity capacity of patients with chronic disease: a cross-sectional study

**DOI:** 10.1038/s41598-022-17047-9

**Published:** 2022-07-22

**Authors:** Edem Allado, Mathias Poussel, Eliane Albuisson, Jean Paysant, Margaux Temperelli, Oriane Hily, Anthony Moussu, Noura Benhajji, Gerôme Gauchard, Bruno Chenuel

**Affiliations:** 1 Université de Lorraine, CHRU-Nancy, University Center of Sports Medicine and Adapted Physical Activity - University Hospital of Nancy, 54000 Nancy, France; 2 Université de Lorraine, DevAH, 54000 Nancy, France; 3 OMEOS, 54000 Nancy, France; 4 CHRU-Nancy, Direction de la Recherche Clinique et de l’Innovation, 54000 Nancy, France; 5 Université de Lorraine, CNRS, IECL, 54000 Nancy, France; 6 Faculté de Médecine, Département du Grand Est de Recherche en Soins Primaires: DEGERESP, Université de Lorraine, 54000 Nancy, France; 7 Rehabilitation Medicine Department, Université de Lorraine, CHRU-Nancy, 54000 Nancy, France

**Keywords:** Physiology, Metabolism

## Abstract

The aim of this study was to evaluate the real intensity level of exercise in a sample of patients with chronic disease from obesity, rheumatology, hematology and other departments involved in a hospital-based program of adapted physical activity (APA). For this cross-sectional study, we studied seventy-five patients with chronic disease and no beta-blocker treatment. They systematically performed a cardiopulmonary exercise test before participating in a supervised APA practice using a telemetry wireless system to monitor heart rate (HR) during the first session. Based upon the results of the functional evaluation of exercise performance, we studied two groups of patients: (1) No limitation in exercise performance (maximal oxygen uptake greater than or equal to 80% of the theoretical reference) and (2) limited exercise performance (maximal oxygen uptake less than 80% of the theoretical value). Fifty-two patients (69.3%) were women, mean age was 42.6 (± 13.8), and mean BMI was 36.7 (± 10.6). Most patients had been referred for obesity (57.3%). We found 39 patients with normal exercise capacities and 36 patients with limited exercise performance. There were no significant differences in demographic and clinical characteristics between the two groups. For all populations, the mean and median real intensity levels of exercise in a sample of patients were moderate (55–70% HR max) and were the same for both groups. During the most intensive 15-min bout of the APA session, the HR for patients in both groups was greater than 70% of the actual maximum HR. This study observed a moderate level of APA exercise intensity in patients suffering from various chronic diseases. We found no significant difference in intensity level of exercise between patients’ capacities, i.e., with and without limitation of their maximal performance.

## Introduction

Sedentary behavior and physical inactivity are major risk factors for non-communicable diseases, not only accounting for 9% of premature mortality but also leading to disability from chronic diseases and a substantial economic burden worldwide^1–3^. It has been demonstrated that active people live 3 years longer than inactive individuals^4,5^.

Sedentary behavior, defined as a low energy expenditure of < 1.5 metabolic equivalent units (MET) in a sitting or reclining posture during waking time, is not only the lowest end of the physical activity broad spectrum, but has also been suggested to be an independent predictor of metabolic risk^1,6,7^. It might be counteracted by regular moderately intensive physical activity (at least 150 min/week of physical activities where energy expenditure is between 3 and 5.9 METs)^8,9^.

In 2004, as a public health priority and to reduce the worldwide problems caused by the deleterious association of physical inactivity and sedentary behavior, the World Health Assembly adopted the WHO global strategy on diet, physical activity and health to promote physical activity. It also endorsed the new Global Action Plan on Physical Activity (GAPPA) 2018–2030, whose target is to reduce global levels of inactivity in adults and adolescents by about 15% by 2030^10,11^.

The new WHO guidelines on physical activity and sedentary behavior were ratified in 2020 and, like previous recommendations, encouraged the integration of exercise counseling in clinical practice, including exercise prescription^12^. In that context, the American College of Sports Medicine (ACSM) launched a multinational initiative called “Exercise is Medicine” to make physical assessment and exercise prescription an integral component of disease prevention and treatment for all patients^13–15^.

In France, the promotion of physical activity was legitimized by the 2016 Health Act of the Ministry of Social affairs and Health which includes the amendment, “prescription of physical activity”, allowing physicians to prescribe physical activity to patients suffering from chronic disease^16,17^. In hospital settings, Adapted Physical Activity (APA) in rehabilitation is usually integrated with physiotherapy and is provided by physiotherapists in conjunction with exercise professionals holding an academic degree in APA^18,19^. The APA program is individualized to the patient and incorporates their physical fitness, exercise preferences, psychological aspects, and expectations. In addition, in the case of hemodialyzed patients, the introduction of APA programs in several countries has shown a positive impact but also shown that it must be included in a global and multidisciplinary approach^20^.

A combined aerobic exercise and resistance-training program is proposed to improve physical fitness, as well as exercises aimed to enhance flexibility and balance. The intensity of exercise should be moderate, providing roughly 55 to 70% of maximum heart rate (HR) or 3 to 5.9 METs and should not be uncomfortable for a patient^21^. Although APA is clearly distinct from rehabilitation, the line between both may sometimes be blurred, especially regarding the intensity of the physical activity performed during a training session.

Our main goal was to evaluate the real intensity level of exercise in a sample of patients suffering from chronic disease following a hospital-based session of the APA program, and to compare this intensity in two groups, those with and without a limitation on exercise performance capacity.

## Methods

This is a cross-sectional monocentric study performed at the Nancy University Center of Sports Medicine and APA, France, between February 2020 and July 2021, in patients requiring implementation of APA. APA is a means that allows the movement of people who, due to their physical, mental, or social status, cannot perform physical activity under normal conditions. The inclusion criteria were age greater than 18 years old, with the ability to perform a cardiopulmonary exercise test and participate in supervised APA practice using a telemetry wireless system to monitor and record HR during the first session. The exclusion criteria were beta-blocker treatment and joint pain or limitations.

### Demographic, clinical data and intervention

Data collected from the included patients were age, gender, and Body Mass Index (BMI). We classified the patient’s referral department categories behind the APA program into four groups: Obesity, Rheumatology, Hematology and Others. The patients' socio-professional status was also categorized: (1) executives and intermediate professions, (2) agricultural workers and artisans, (3) blue collar/manual workers (4) retirees, unemployed, others, (5) students and (6) incapacitated through ill health. A maximum cardiopulmonary exercise test was carried out on all included patients.

Following the cardiopulmonary exercise test, the sample was split into two groups^22–25^: (1) No limitation of exercise performance capacities: those with normal maximal oxygen uptake ([V′O_2_peak] greater than or equal to 80% of the reference value) and (2) limitation of exercise performance capacities defined by a reduced V′O_2_peak (less than 80% of the reference value). We used the Wasserman equation to determine reference values for capacities assessment^22,26,27^. The actual peak HR (HRpeak) was measured during the exercise test. We collected the VT1 first ventilatory threshold (VT1) in each exercise test. VT2 (second ventilatory threshold) could not be collected because it did not reach the patients.

- Exercise test procedure: V′O_2_max was established during an incremental exhaustive exercise test on a cycle ergometer (eBike, GE Healthcare, France). For each patient, a progressively increasing work rate protocol was designed (rest, followed by an incremental phase of exercise every minute) so that volitional exhaustion is reached following 8–12 min of exercise. Respiratory and metabolic variables (minute-ventilation, tidal volume, frequency of breathing, V′O_2_, V′CO_2_) were measured breath-by-breath through a mask connected to a pneumotachograph and O_2_ and CO_2_ analyzers (Vyntus™ CPX, Vyaire, Germany). Criteria for the achievement of V′O_2_max were HR > 90% of the maximal reference value HR (220—age), respiratory exchange ratio ([RER] = V′CO_2_/V′O_2_) > 1.1 or V’O_2_ plateau^22,23,28^. The effort consisted of going up to the patient’s maximum capacity and was followed upon with a 6-min recovery phase to reach the resting state back.

The effort intensity levels are defined as such: sedentary: < 40% HR max or > 1.5METs, low: 40–55% HR max or 1,6 et 3 METs; moderate: 55 to 70% HR max or 3 and < 6 METs and high: 70 to 90% HR max or 6 to < 9 METs^29^.

During the APA session, HR was digitized at 1 Hz (using custo guard, Custo Med GmbH). The ratio between the APA session HR and exercise test HRpeak was used to assess the intensity of effort.

#### The hospital-based APA intervention

The APA program comprised two hospital-based APA interventions per week, supervised by a physiotherapist and a trained APA educator (exercise professionals). The typical session lasted 60 min with 20 min of constant aerobic effort on an ergocycle at a comfortable intensity level. Subsequently, a 20-min body weight reinforcement session, consisting of 1-min effort followed by 1–2-min recovery phases were performed. At the end of the session, a 10-min stretching time was planned. The intensity was individually set to correspond to moderate exercise (55 to 70% of maximum heart rate (HR), 3 to 5.9 és), where participants reached a comfortable level of exercise, following the speech rule which defined the maximum perceived exertion. The program was organized in two stages: 10 to 15 min of aerobic activity for warm-up then 30 min of strengthening activity in interval training of a maximum of 6 sets of a maximum of one minute.

### Statistical analysis

Both descriptive and comparative analyses were made by accounting for the nature and distribution of the variables. Qualitative variables were described as frequencies and percentages; quantitative variables were evaluated using the mean ± standard deviation (SD) or with the median and interquartile range (IQR). The chi-square test or Fisher’s exact test with, if necessary, the exact calculation of Fisher, was used for the ordinal or nominal data analysis. We use the student T test to compare age, BMI, HR. The significance level was set at 0.05 for the entire study. IBM SPSS Statistics 23.0 was used for the data analysis.

### Ethics and dissemination

All data used were obtained from the medical records. No supplementary examination was necessary for patients to meet the inclusion criteria. This study is registered with the Information Technology and Freedoms Commission for the University Hospital of Nancy (file number: 2021PI191) and on Clinicaltrials.gov (NCT05146544) and was designed in accordance with the general ethical principles outlined in the Declaration of Helsinki. This study has received approval from the French Ethics Committee (*Comité d’éthique CHRU de Nancy, saisine n°336 01/12/2021*). Informed consent was obtained from all subjects and their legal guardians. All patients gave their consent for the use of their medical data during the period they received medical care at the University Hospital.

## Results

Seventy-five patients were included, 69.3% (n = 52) were women, mean age was 42.6 (± 13.8) and mean BMI was 36.7 (± 10.6) Kg/m^2^. Most patients were obese (57.3%) or had rheumatological disease (13.3%). Blue-collar/manual workers, executives/intermediate positions and retirees formed 56.0%, 18.7% and 16.0%, respectively, of the sample. Mean of HR_peak_ and V′O_2peak_ were 153.9 (20.9) bpm and 17.2 (4.9) mL/min/kg respectively. HR_peak_ is about 87.0% of age predicted HR_max_. Sixty-seven patients had RER greater than or equal to 1. Among this sample, fifty patients achieved VO_2max_. We categorized 39 (52.0%) and 36 (48.0%) of patients as having no limited (*greater* than or equal to 80% of the reference value) or limited (*less* than 80% of the reference value) exercise performance, respectively. There were no significant differences between the two categories for demographic and clinical characteristics (Table 1).

**Table 1:** Baseline demographic and clinical characteristics (n = 75).

	Total (n = 75)	No limitation of exercise performance (n = 39)	Limited exercise performance (n = 36)	p-value*
Women	52 (69.3)	30 (76.9)	22 (61.1)	0.137
Age, years	42.6 (13.8)	44.9 (13.8)	40.0 (13.6)	0.129
Body mass index, kg/m^2^	36.7 (10.6)	35.8 (10.2)	37.7 (11.0)	0.758
V’O_2peak_, ml/min/kg	17.2 (4.9)	19.4 (4.8)	14.9 (4.1)	**0.001**
**HR**_**peak**_				0.079
Value (mean (SD))	153.9 (20.9)	157.9 (21.2)	149.5 (19.8)	
HR_peak_ > 90% x HR_max_ (n(%))	28 (37.3)	19 (48.7)	9 (25)	
**RER**_**peak**_				0.401
Value (mean (SD))	1.11 (0.11)	1.12 (0.10)	1.10 (0.12)	
RER_peak_ > 1.1 (n (%))	43 (57.3)	23 (59.0)	20 (55.6)	
**First ventilatory threshold**				0.404
HR(VT_1_)	123.5 (15.8)	125.0 (15.8)	121.9 (15.9)	
HR(VT_1_)/HR_peak_	80.8%	79.6%	82.1%	
**First ventilatory threshold**				**0.001**
V’O_2_(VT_1_) (ml/min/kg)	11.9 (3.8)	13.5 (3.8)	10.1 (3.1)	
V’O_2_(VT_1_)/VO2_peak_	70.0¨%	71.3%	68.6%	
**Pathologies**				0.581
Obesity	43 (57.3)	21 (53.8)	22 (61.1)	
Rheumatology	10 (13.3)	4 (10.3)	6 (16.7)	
Hematology and oncology	6 (8.0)	5 (12.8)	1 (2.8)	
Diabetology	2 (2.7)	1 (2.6)	1 (2.8)	
Pneumology	1 (1.3)	1 (2.6)	0 (0.0)	
Internal Medicine	3 (4.0)	1 (2.6)	2 (5.6)	
Other (immunology, nephrology)	10 (13.3)	6 (15.4)	4 (11.1)	
**Socio-professional status**				0.318
Executives, intermediate professions	14 (18.7)	7 (17.9)	7 (19.4)	
Agricultural/artisan workers	3 (4.0)	0 (0.0)	3 (8.3)	
Blue collar/manual workers	42 (56.0)	23 (59.0)	19 (52.8)	
Retirees, unemployed, others	12 (16.0)	5 (12.8)	7 (19.4)	
Students	0 (0.0)	0 (0.0)	0 (0.0)	
Incapacitated through ill health	1 (1.3)	1 (2.6)	0 (0.0)	
Data missing	3 (4.0)	3 (7.7)	0 (0.0)	

Significant values are in bold.

Data are presented as n (%) for dichotomous variables, mean (SD) for continuous demographic variables.

*We use the student T test to compare age and body mass index, and chi-square test or Fisher’s exact test for other variables.

The 60-min APA session HR average was 105.3 (± 16.6) bpm which represents 69.2% of HRmax. There was a significant difference between the most intense 30 min, which was 105.2 (± 17.5) bpm represent 69.1% and the most intense 15 min, which was 115.5 (± 19.4) bpm, which represents 75.8% of HRpeak (p < 0.001). We observed higher HR in patients with limited exercise performance with HR over 115.6 (± 20.3) bpm, representing 77.8% of HRpeak over the most critical 15 min (p = 0.996). However, we found no significant difference between patients with normal and those with limited exercise performance in the three periods overall (Table 2).

**Table 2:** Adapted physical activity heart rate and intensity of patient exercise capacity.

	Total (n = 75)	No limitation of exercise performance (n = 39)	Limited exercise performance (n = 36)	p-value*
**Full APA session (bpm)**				
HR (bpm)	105.3 (16.6)	104.3 (15.4)	106.4 (18.0)	0.597
HR/HR_peak_	69.2%	66.9%	71.6%	0.076
HR/HR_VT1_	85.8%	84.1%	87.7%	0.246
**30 min most intensive (bpm)**				
HR (bpm)	105.2 (17.5)	104.6 (17.6)	105.9 (17.6)	0.750
HR/HR_peak_	69.1%	67.0%	71.3%	0.110
HR/HR_VT1_	85.7%	84.2%	87.4%	0.322
**15 min most intensive (bpm)**				
HR (bpm)	115.5 (19.4)	115.6 (18.8)	115.6 (20.3)	0.996
HR/HR_peak_	75.8%	74.0%	77.8%	0.192
HR/HR_VT1_	94.1%	93.0%	95.3%	0.191

Data are presented as mean (SD) for continuous variables.

*We use the student T test to compare heart rate between no limitation of exercise performance and limited exercise performance.

## Discussion

The mean and median real intensity level of exercise of hospital-based sessions of APA in a sample of patients was moderate (69.2% of maximal capacity). Only the most intensive 15 min produced an intensified level (75.8% of maximal capacity). In our sample of patients suffering from different chronic diseases, we found no significant statistical difference in maximal intensity during hospital-based APA sessions between subjects with and without performance limitation.

In theory, the maximal level of exertion for moderate physical activity corresponded to a comfortable effort completely in the group without any cardiopulmonary limitation, but the level of exercise for APA slightly exceeded the conventional target of 55–69% of HRpeak, achieving moderate exercise in performance limited patients (Table 2).

These results emphasize the need for precautionary functional assessment of cardiopulmonary fitness in fragile chronic disease patients prior to any exercise prescription since acute, vigorously intense exercise is well known to increase cardiovascular events^30^. Nevertheless, in order to include them in the global healthcare approach and get them supported, it is essential to recognize the role of exercise professionals. Our results therefore highlight the key role of healthcare professionals, not only to promote regular physical activity in patients but also to rigorously assess their health status. Indeed, based on accurate identification of specific groups of patients, taking into account patient preferences, and providing written physical activity programs, public health policy regarding physical activity on prescription has been shown to be effective in increasing the level of physical exercise in patients^31,32^. Throughout patient care, and in order to support a change to a more autonomously active and less sedentary lifestyle, healthcare professionals generally focus on patient-centred dialog, individually tailored physical activity recommendations with a written prescription, and follow-up. Because of the increased levels of stress during physical exercise, our results support the idea that healthcare professionals should meticulously evaluate the patient’s health status. This will undoubtedly enhance the effectiveness of the 2018–2030 WHO GAPPA slogan—“More active people for a healthier world”.

To reduce the potential risk of injury, elderly or debilitated patients should receive moderate intensity exercise prescription in preference to ideally moderate physical activity. It could be explained by a physiologic aging reduction induced by a regular practice. Ekelund et al. have recently demonstrated a dose–response association between reducing substantial risk and mildly intensive physical activity^33^. As previously noted by Blair et al., “a little is better than none” is an important public health message in the promotion of health-enhancing physical activity^34^. Moreover, a higher level of total physical activity, regardless of intensity level, associated with less time spent sedentary, decreases the risk of premature mortality^33^. In heart failure patients with preserved ejection fraction, high intensity interval training or continuous moderate training did not change peak oxygen uptake compared to control of guideline-based physical activity^35^. In the peculiar context of fragile or elderly patients, the interest of resistance training needs to be highlighted. Indeed, the benefical role of strength training in the reduction of falls in the elderly has been demonstrated, and has been more recently observed to lower mortality, with an additive effect when combined with dynamic exercise^36–38^.

This study has limitations. Firstly, the size sample prevents any extension of the results. In fact, the study was performed in a small sample (n = 75) practicing APA in a single hospital center. In addition, the presence of a large number of obese participants limited a subgroup exploration of different chronic pathologies. Therefore, although HR is the most convenient variable for evaluating the intensity in ecologic condition, it presents limitations. In fact, it is subject to numerous artefacts and the HR curve is influenced by age, gender, and performance^39^.

The main strength of this study is its originality, as we could record the actual practice intensity in APA sessions from physiological data recorded. We use for this study incremental exercise test and the ability to individual capacities. Indeed, VO2max as a gold standard to assess the functional capacities of patients. This study also incorporates all the recommendations of the Health Act of the Ministry of Social Affairs and Health.

In conclusion, our study explored a high level of exercise intensity (> 70% of HRpeak in exercise tests) in patients suffering from different chronic diseases, with and without performance limitation. Our results emphasize that training at the perceived exertion level could produce an efficient level of exercise to improve health. However, an analysis of a larger population in a longitudinal study would make this observation more explicit. This would also open up the possibility of creating personalized programs appropriate to the health status of the patient so that medical risk can be evaluated and an appropriate safe exercise program developed.

## Data Availability

The datasets used and/or analysed during the current study available from the corresponding author on reasonable request.

## Abbreviations

APA: Adapted physical activity
BMI: Body mass index
HR: Heart rate
HRpeak: Peak heart rate
IQR: Interquartile range
RER: Respiratory exchange ratio
SD: Standard deviation
V′O_2_max: Maximal oxygen uptake
V′O_2_peak: Oxygen uptake peak
VT1: First ventilatory threshold
